# Effects of Chronic Intracerebroventricular Infusion of RFamide-Related Peptide-3 on Energy Metabolism in Male Mice

**DOI:** 10.3390/ijms21228606

**Published:** 2020-11-15

**Authors:** Shogo Moriwaki, Yuki Narimatsu, Keisuke Fukumura, Eiko Iwakoshi-Ukena, Megumi Furumitsu, Kazuyoshi Ukena

**Affiliations:** Laboratory of Neurometabolism, Graduate School of Integrated Sciences for Life, Hiroshima University, Higashi-Hiroshima, Hiroshima 739-8521, Japan; m203300@hiroshima-u.ac.jp (S.M.); m192783@hiroshima-u.ac.jp (Y.N.); kfuku@hiroshima-u.ac.jp (K.F.); iwakoshi@hiroshima-u.ac.jp (E.I.-U.); mfurumi@hiroshima-u.ac.jp (M.F.)

**Keywords:** neuropeptide, RFamide-relative peptide, food intake, body mass, body temperature

## Abstract

RFamide-related peptide-3 (RFRP-3), the mammalian ortholog of avian gonadotropin-inhibitory hormone (GnIH), plays a crucial role in reproduction. In the present study, we explored the other functions of RFRP-3 by investigating the effects of chronic intracerebroventricular infusion of RFRP-3 (6 nmol/day) for 13 days on energy homeostasis in lean male C57BL/6J mice. The infusion of RFRP-3 increased cumulative food intake and body mass. In addition, the masses of brown adipose tissue (BAT) and the liver were increased by the administration of RFRP-3, although the mass of white adipose tissue was unchanged. On the other hand, RFRP-3 decreased O_2_ consumption, CO_2_ production, energy expenditure, and core body temperature during a short time period in the dark phase. These results suggest that the increase in food intake and the decrease in energy expenditure contributed to the gain of body mass, including the masses of BAT and the liver. The present study shows that RFRP-3 regulates not only reproductive function, but also energy metabolism, in mice.

## 1. Introduction

Reproductive function is related to the metabolic status and energy reserves of an organism [1]. It is known that sufficient fat storage in the body is necessary for the onset and maintenance of menstrual cycles [2]. Systemic energy homeostasis is affected by the coordinated regulation of energy intake and expenditure. Energy intake exceeding energy expenditure leads to a chronic positive energy balance, storage of excess energy in the adipose tissues, and subsequent obesity [3]. Energy metabolism, including feeding behavior, is regulated by hypothalamic neuropeptides and peripheral hormones. Among them, neuropeptide Y (NPY)/agouti-related peptide (AgRP) neurons increase food intake and decrease energy expenditure, whereas proopiomelanocortin (POMC) neurons have the opposite effect [4]. Peripheral hormones, such as orexigenic ghrelin and anorexigenic leptin, act on these hypothalamic neurons and regulate feeding behavior [5,6]. In addition to energy homeostasis, the *ob/ob* mouse, lacking a functional leptin gene, is infertile and has atrophic reproductive organs [7].

In vertebrates, the reproductive system is controlled by the hypothalamic–pituitary–gonadal (HPG) axis, in which gonadotropin-releasing hormone (GnRH) secretion from the hypothalamus stimulates the production of luteinizing hormone (LH) and follicle-stimulating hormone (FSH) from the pituitary gland [8,9]. Recently, two neuropeptides of the RFamide family have been identified as important regulators of the HPG axis. The first one, kisspeptin, is a stimulator of GnRH secretion, and seems to be the principal conduit for mediating sex steroid feedback [10]. It has been demonstrated that kisspeptin affects the energy balance [11]. The second one, gonadotropin-inhibitory hormone (GnIH), has been discovered as the first avian hypothalamic peptide that inhibits gonadotropin release in quails [12]. After the discovery of GnIH in birds, GnIH orthologs were found in a number of other vertebrates, from fish to humans [13,14,15]. We also identified mature endogenous RFamide-related peptide (RFRP), a mammalian ortholog of GnIH, in the hypothalamus of rats [16]. GnIH and its orthologs, including RFRP, have a common *C*-terminal Leu-Pro-Xaa-Arg-Phe-NH_2_ (Xaa = Leu or Gln) motif [17,18]. The precursor gene, *Rfrp*, encodes a precursor that produces two peptides, RFRP-1 and RFRP-3, in mammals [14,16,19,20,21,22]. Early research showed that intracerebroventricular (i.c.v.) injection of RFRP-1 increased prolactin release in rats [20], and a large body of evidence now indicates that LH secretion is inhibited by RFRP-3 in various mammalian species [14,19,23,24,25,26,27,28,29,30,31]. The receptor for RFRP is G protein-coupled receptor 147 (GPR147), which is also called neuropeptide FF receptor 1 and OT7T022, in mammals [20]. This receptor is highly expressed in the hypothalamus and gonads of rats [20,32] and mice [33]. Some reports have suggested that food intake was stimulated by acute i.c.v. injection of GnIH/RFRP-3 in chickens and rats [25,28,34,35]. In addition, a recent study showed that chronic intraperitoneal injection of RFRP-3 had an orexigenic effect and regulated glucose homeostasis in rats [36]. Therefore, it is possible that RFRP not only influences reproductive functions, but may also regulate energy homeostasis in birds and mammals.

However, little is known about the functions of RFRP-3 in mice in comparison with other mammals, because the mature endogenous RFRP-1 and RFRP-3 peptides had not been identified in mice until recently [37]. We previously found that RFRP-containing perikarya were distributed in the dorsomedial hypothalamus (DMH) in mice, and the RFRP-containing fibers were projected to the arcuate nucleus (Arc) and paraventricular nucleus (PVN) by immunohistochemical analysis [38]. It is well known that these regions are involved in energy metabolism, including feeding behavior [39]. In previous studies, the central actions of RFRP on energy metabolism, including body mass gain, energy expenditure, and body temperature, were not revealed in any animals. In this study, we investigated the effect of chronic i.c.v. infusion of RFRP-3 on the regulation of energy metabolism in mice.

## 2. Results

### 2.1. Effects of Chronic I.C.V. Infusion of RFRP-3 on Food Intake and Body Mass

**Figure 1:**
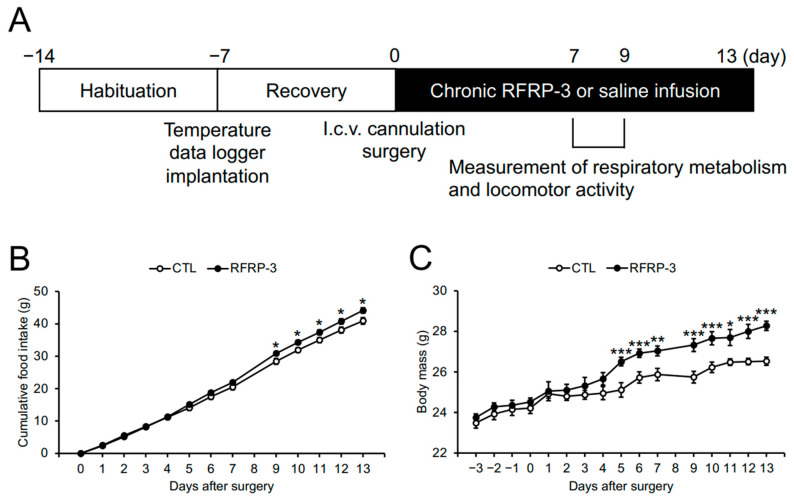
The effects of chronic intracerebroventricular (i.c.v.) infusion of RFamide-related peptide-3 (RFRP-3) on food intake and body mass: (**A**) experimental schedule, (**B**) cumulative food intake, and (**C**) body mass. The food intake and body mass could not be measured 8 d after the infusion of RFRP-3 because the mice were housed in a metabolic cage. Each value represents the mean ± standard error of the mean. *n* = 7–8; * *p* < 0.05, ** *p* < 0.01, *** *p* < 0.005.

To investigate whether RFRP-3 affects energy homeostasis in mice, we performed a 13-day chronic i.c.v. infusion of RFRP-3 (6 nmol/day) using osmotic pumps, and measured their food intake and body mass. The experimental schedule is shown in Figure 1A. Cumulative food intake and body mass were increased by the infusion of RFRP-3 (Figure 1B,C).

### 2.2. Effects of Chronic I.C.V. Infusion of RFRP-3 on Body Composition

**Figure 2:**
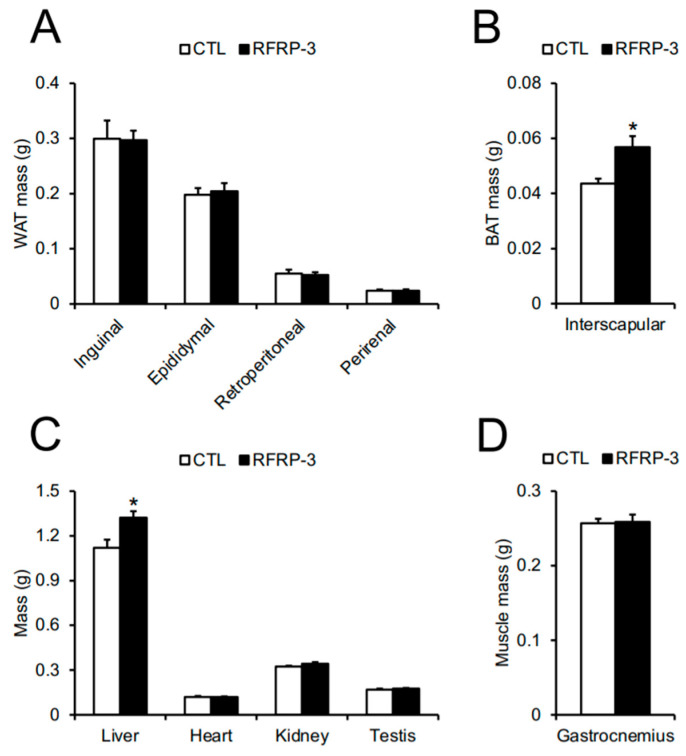
The effects of chronic intracerebroventricular (i.c.v.) infusion of RFamide-related peptide-3 (RFRP-3) on body composition. (**A**) Inguinal, epididymal, retroperitoneal, and perirenal white adipose tissue (WAT) mass. (**B**) Interscapular brown adipose tissue (BAT) mass. (**C**) Liver, heart, kidney, and testis mass. (**D**) Gastrocnemius muscle mass. Each value represents the mean ± standard error of the mean. *n* = 7–8; * *p* < 0.05.

To examine what contributed to the body mass gain in chronic RFRP-3 infused mice, we weighed the adipose tissues, organs, and muscle. Although the masses of the inguinal, epididymal, retroperitoneal, and perirenal white adipose tissues (WAT) were unchanged (Figure 2A), the mass of the interscapular brown adipose tissue (BAT) increased (Figure 2B). In addition, the liver mass increased, while the masses of the heart, kidney, testis, and gastrocnemius muscle tissues did not change (Figure 2C,D).

Because the masses of BAT and the liver were increased through the infusion of RFRP-3, we evaluated the serum levels of the glucose, lipids, and insulin. The serum glucose level was increased by the infusion of RFRP-3, while no change in the serum levels of the lipids (free fatty acids, triglyceride, and cholesterol) and insulin was observed (Table 1).

Our aim was to investigate the effects of chronic i.c.v. infusion of RFRP-3 on the expression of the central neuropeptides and hormones involved in feeding behavior, energy metabolism, and reproduction. Therefore, we also analyzed the transcriptional levels of the represented neuropeptides and hormones related to their functions in the hypothalamus and pituitary by qRT-PCR. These factors were as follows: NPY, AgRP, and POMC as neuropeptides related to feeding; thyrotropin-releasing hormone (TRH), growth hormone (GH), and thyroid-stimulating hormone β (TSHβ) as neuropeptides and hormones related to energy metabolism; GnRH, prolactin (PRL), LHβ, and FSHβ as neuropeptides and hormones related to reproduction; and RFRP, and GPR147 as the receptor for RFRP. The mRNA expression levels were not changed by the infusion of RFRP-3 (Figure S1A,B).

On the other hand, to investigate the cause of the mass gains in BAT and the liver by the infusion of RFRP-3, we analyzed the expression of several enzymes and related factors (i.e., lipogenesis and lipolysis) in the BAT, liver, and inguinal WAT (iWAT) using qRT-PCR. These factors were as follows: acetyl-CoA carboxylase (ACC), fatty acid synthase (FAS), stearoyl-CoA desaturase 1 (SCD1), and glycerol-3-phosphate acyltransferase 1 (GPAT1) as lipogenic enzymes; carbohydrate-responsive element-binding protein α, β (ChREBPα,β) as lipogenic transcription factors; carnitine palmitoyltransferase 1a (CPT1a), adipose triglyceride lipase (ATGL), and hormone-sensitive lipase (HSL) as lipolytic enzymes; peroxisome proliferator-activated receptor α, γ (PPAR α, γ) as lipid-activated transcription factors; glyceraldehyde-3-phosphate dehydrogenase (GAPDH) as a carbohydrate metabolism enzyme; cluster of differentiation 36 (CD36) as a fatty acid transporter; phosphoenolpyruvate carboxykinase (PEPCK) and glucose-6-phosphatase (G6Pase) as gluconeogenesis enzymes; solute carrier family 2 member 2 (SLC2A2) and SLC2A4 as glucose transporters; and tumor necrosis factor α (TNF α) as an inflammatory cytokine. The qRT-PCR data show that the chronic i.c.v. infusion of RFRP-3 did not affect the transcriptional levels of these factors (Figure S2A–C).

### 2.3. Effects of Chronic I.C.V. Infusion of RFRP-3 on Respiratory Metabolism, Locomotor Activity, and Core Body Temperature

**Figure 3:**
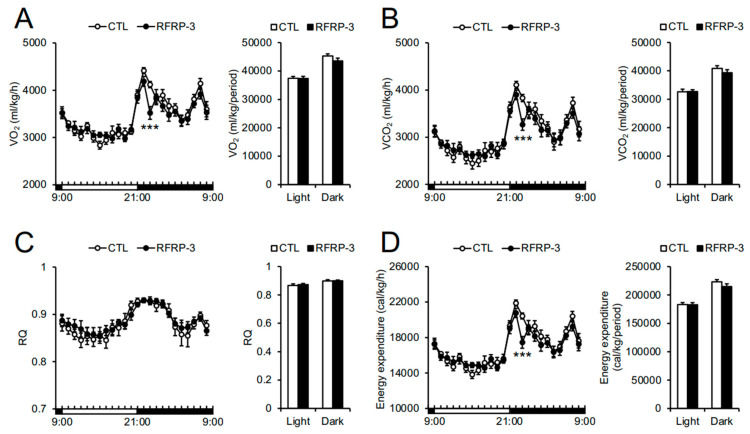
The effects of chronic intracerebroventricular (i.c.v.) infusion of RFamide-related peptide-3 (RFRP-3) on O_2_/CO_2_ metabolism hourly. (**A**) O_2_ consumption (VO_2_) measured in the metabolic cage. (**B**) CO_2_ production (VCO_2_) measured in the metabolic cage. (**C**) The respiratory quotient (RQ) measured in the metabolic cage. (**D**) Energy expenditure calculated by VO_2_ and VCO_2_. Each value represents the mean ± standard error of the mean. *n* = 7–8; *** *p* < 0.005.

**Figure 4:**
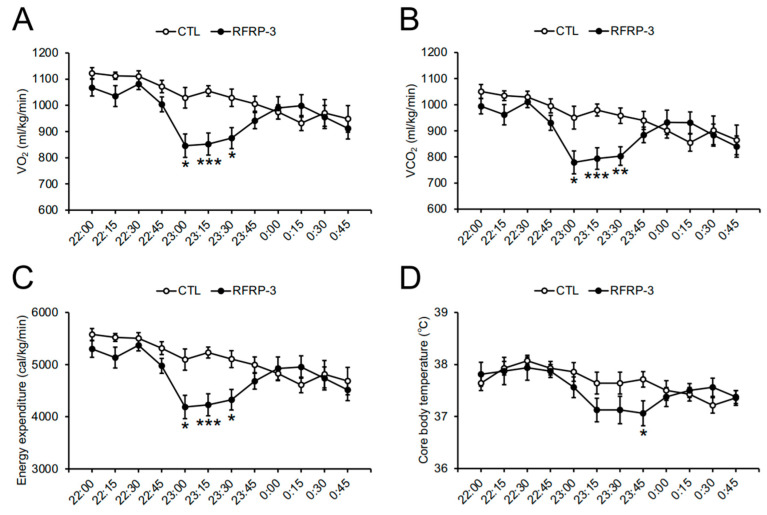
The effects of chronic intracerebroventricular (i.c.v.) infusion of RFamide-related peptide-3 (RFRP-3) on O_2_/CO_2_ metabolism and core body temperature every 15 min. (**A**) O_2_ consumption (VO_2_) measured in the metabolic cage. (**B**) CO_2_ production (VCO_2_) measured in the metabolic cage. (**C**) Energy expenditure calculated by VO_2_ and VCO_2_. (**D**) The core body temperature measured by the implanted temperature data logger. Each value represents the mean ± standard error of the mean. *n* = 7–8; * *p* < 0.05, ** *p* < 0.01, *** *p* < 0.005.

**Figure 5:**
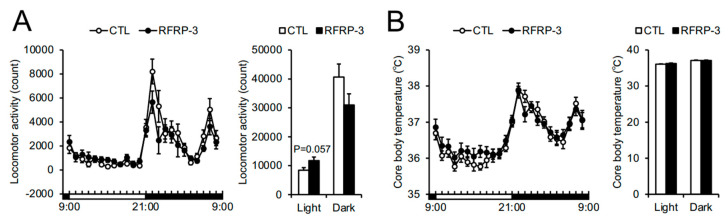
The effects of chronic intracerebroventricular (i.c.v.) infusion of RFamide-related peptide-3 (RFRP-3) on locomotor activity and core body temperature hourly. (**A**) The spontaneous locomotor activity measured by an infrared ray passive sensor. (**B**) The core body temperature measured by the implanted temperature data logger. Each value represents the mean ± standard error of the mean *n* = 7–8.

Next, we measured O_2_ consumption (VO_2_) and CO_2_ production (VCO_2_) 8 days after RFRP-3 infusion so as to analyze whole-body respiratory metabolism. The VO_2_, VCO_2_, and energy expenditure over a short time period in the dark phase (23:00 p.m.–00:00 a.m.) were decreased by the infusion of RFRP-3, although the respiratory quotient (RQ) ratio remained unchanged (Figure 3A–D). When we conducted a detailed analysis of the respiratory metabolism every 15 min, the VO_2_, VCO_2_, and energy expenditure between 23:00 p.m. and 23:45 p.m. decreased through the infusion of RFRP-3 (Figure 4A–C). In addition, the locomotor activity showed no change 8 days after RFRP-3 infusion (Figure 5A). In order to examine the effects of the infusion of RFRP-3 on thermogenesis in the whole body, we measured the core body temperature following RFRP-3 infusion. The core body temperature did not change hourly after the infusion of RFRP-3 (Figure 5B). When a detailed analysis of core body temperature was conducted every 15 min, a decline in the core body temperature in the dark phase (23:45 p.m.) was found (Figure 4D).

To assess the possible influences on the serum thyroid hormone levels related to energy metabolism, we measured the serum thyroxine (T4) level. The serum T4 level remained unchanged (Table 1). In addition, we analyzed the mRNA expression levels of the genes related to the thermogenic function in BAT using qRT-PCR. These factors were as follows: peroxisome proliferator-activated receptor γ coactivator 1α (PGC1α), uncoupling protein 1 (UCP1), type II iodothyronine deiodinase (DIO2), cell death-inducing DNA fragmentation factor-like effector A (CIDEA), and cytochrome c oxidase subunit 4 (COX4). qRT-PCR showed that the transcriptional levels of these factors were unchanged by the infusion of RFRP-3 (Figure S2A).

## 3. Discussion

After our first identification of mature endogenous RFRP-3 from the rat hypothalamus in 2002 [16], RFRP has been recognized as a neuropeptide involved in mammalian reproduction [14,19,23,24,25,26,27,28,29,30,31]. Subsequently, some reports have shown that the acute i.c.v. injection of GnIH/RFRP-3 increased food intake in chicks [34,35], mice [40], rats [25,28], sheep [40], and cynomolgus monkeys [40]. In addition, a recent study showed that chronic intraperitoneal injection of RFRP-3 to rats stimulated feeding behavior and regulated blood glucose homeostasis [36]. However, the chronic central effects of RFRP-3 have not been elucidated in any animals. In the present study, we investigated the effect of chronic i.c.v. infusion of mouse RFRP-3 on energy metabolism in mice for the first time. These data indicate that RFRP-3 increased food intake, blood glucose level, and body mass gain, including the masses of BAT and liver in mice. Furthermore, we observed temporal decreases in energy expenditure and core body temperature for a short period in the dark phase, i.e., active phase of mice.

A significant increase in body mass was observed from 5 days after the infusion of RFRP-3, although a significant increase in cumulative food intake was observed from 9 days after the infusion of RFRP-3 (Figure 1B,C). These results suggest that body mass may be independent of food intake and may be related to the decrease in energy expenditure. It has been recently reported that intraperitoneally injected RFRP-3 slightly increased body mass and serum lipid levels in rats [36]. Although the serum glucose level was increased by the infusion of RFRP-3 in this study, no change in the serum lipid and insulin levels was found (Table 1). This result suggests the presence of differences in the species or target sites of RFRP-3 by using rats/mice and intraperitoneal or central injections.

The chronic i.c.v. infusion of RFRP-3 also increased the masses of BAT and the liver (Figure 2B,C). The increase in body mass was larger than that in the masses of BAT and the liver by the infusion of RFRP-3. Therefore, it was not clear which part(s) of the body contributed to body mass gain in the present study. The cause for body mass gain by RFRP-3 requires further investigation of the whole-body composition. In addition, there were no changes in the mRNA expression levels of the lipogenic and lipolysis-related factors in the BAT, liver, and iWAT (Figure S2). Furthermore, the transcriptional levels of the genes involved in the thermogenic function did not change in the BAT (Figure S2A). It is necessary to analyze the translational levels of these factors in the future study. It has been reported that hepatic fat accumulation is induced by an excessive increase in the mass of WAT or serum lipid levels [41]. In the present study, the mass of the WAT and serum lipid levels were unchanged by the infusion of RFRP-3 (Figure 2A and Table 1). Hepatic triglyceride and histological staining in order to discover the cause of the increased liver mass by the chronic i.c.v. infusion of RFRP-3 remain to be examined in the liver. It is well known that BAT thermogenesis is deeply involved in energy expenditure [42,43,44]. In this study, the infusion of RFRP-3 increased the mass of BAT and decreased the energy expenditure and core body temperature (Figure 2B and Figure 4C,D). It has been reported that the mass of BAT is increased by mitochondrial dysfunction and lipid droplet accumulation [45]. Thus, the chronic i.c.v. infusion of RFRP-3 may induce the functional depression of BAT thermogenesis, and eventually increase the mass of BAT because of lipid accumulation. Future studies are necessary in order to investigate the relationship between fat deposition and increased masses in the BAT induced by RFRP-3.

Neuropeptides related to feeding behavior can be involved in the respiratory metabolism [3]. The present study showed that the VO_2_, VCO_2_, and energy expenditure were decreased by the infusion of RFRP-3 during a short time period (around 23:00–23:45 p.m.) in the dark phase (Figure 3A,B,D and Figure 4A–C). The temporal decrease in the core body temperature may have occurred after the decrease in energy expenditure (Figure 4). This is the first evidence that the i.c.v. infusion of RFRP-3 decreased energy expenditure in mice. RFRP-3 neurons are regulated by a photoperiod through the melatonin signal in quails and hamsters [46,47,48]. On the other hand, RFRP neuron activation is independent of the circadian rhythm in male mice [49]. In the present study, it is possible that the effect of the infusion of RFRP-3 on the respiratory metabolism was not influenced by melatonin signals because the C57BL/6J mice used in this study are a melatonin-deficient strain [50]. In addition, mice are nocturnal animals and actively consume food in the early dark phase. These findings suggest that the effect of RFRP-3 on the respiratory metabolism, including energy expenditure, may be affected by changes in energy conditions depending on feeding. Future studies are needed in order to analyze the influence of RFRP-3 on energy metabolism under restricted feeding in male mice.

To examine how RFRP-3 affects the mechanism of regulation of feeding and energy metabolism, we analyzed the mRNA expression levels of the represented neuropeptides and hormones related to feeding and energy metabolism in the hypothalamus and pituitary. These transcriptional levels were not changed by the infusion of RFRP-3 in the hypothalamus and pituitary (Figure S1A,B). Future studies are necessary in order to analyze the translational and/or secretory levels of these factors. It has been reported that GnIH/RFRP stimulates NPY neurons in chicks [34], rats, and sheep [40]. POMC neurons are activated by GnIH/RFRP in sheep, but not rats [40], although the mRNA expression of POMC is decreased in chicks [34]. In addition, RFRP neurons are located in the DMH, an important region in feeding and thermogenesis, and the RFRP-containing fibers projected to the Arc and PVN in mice [37,38]. These fibers are in close contact with the NPY and POMC cells in the Arc of mice [51,52]. Furthermore, the electrophysiological data also show that RFRP inhibits the neuronal activities of the NPY and POMC cells in mice [51,52]. This effect of RFRP on the POMC cells is at least one of the potential mechanisms mediating the orexigenic action by RFRP-3 in mice [51]. The relationship between RFRP and NPY is complicated because the inhibition of NPY cells by RFRP is not consistent with the increase in food intake [52]. In another aspect, some neurons in the DMH activate the sympathetic premotor neurons controlling thermogenesis in BAT [53]. RFRP-3 may directly affect thermogenesis via neurons in the DMH of mice. In future studies, it will be critical to investigate the target cells of RFRP-3-containing neurons so as to regulate energy metabolism, including energy expenditure and thermogenesis, in mice. In contrast, the present study showed that the mass of the testes did not change (Figure 2C) and the *Lhβ* mRNA expression level remained unchanged in the pituitary (Figure S1B). It has been demonstrated that RFRP-3 regulates the serum LH levels in quail [54], rats [28], Syrian hamster [55], and mice [56]. These findings suggest that the chronic i.c.v. infusion of RFRP-3 could not exert its function in reproduction in male mice.

In summary, the present study is the first report showing the chronic central effect of RFRP-3 on the regulation of energy metabolism, including orexigenic action, in mice. Furthermore, the administration of RFRP-3 in the brain regulated systemic energy balance through temporal decreases in energy expenditure and core body temperature during the early dark phase. Further studies are needed in order to understand the molecular and/or anatomical mechanisms of regulation by RFRP-3 in energy metabolism.

## 4. Materials and Methods

### 4.1. Animals

Male C57BL/6J mice (7 weeks old) were purchased from Japan SLC (Shizuoka, Japan) and were housed under standard conditions (25 ± 1 °C under a 12 h light/dark cycle) with ad libitum access to water and normal chow (CE-2; CLEA Japan, Tokyo, Japan). The experimental schedule is shown in Figure 1A.

All of the animal experiments were performed according to the Guide for the Care and Use of Laboratory Animals prepared by Hiroshima University (Higashi-Hiroshima, Japan) and these procedures were approved by the Institutional Animal Care and Use Committee of Hiroshima University (permit number: C19-8, 30 August 2019).

### 4.2. Production of RFRP-3

RFRP-3 containing 10 amino acid residues (SHFPSLPQRF-NH_2_), identical to the mouse RFRP-3 sequence [37], was synthesized by microwave-assisted solid-phase peptide synthesis using an automated peptide synthesizer (Syro Wave; Biotage, Uppsala, Sweden), as previously described [57].

### 4.3. Measurement of Core Body Temperature

The temperature data logger (Thermochron SL; KN Laboratories, Osaka, Japan) was implanted interperitoneally in 8-week-old mice. The mice were left to recover for 1 week before the start of the experiment.

Their core body temperatures were measured every 15 min with ±0.5 °C accuracy. The data were collected hourly or every 15 min for 8 days after the infusion of RFRP-3.

### 4.4. Chronic I.C.V. Infusion

After recovery from the logger implantation, for the 13-day chronic i.c.v. infusion of RFRP-3, the infusion cannula (28 gauge, 328OP; Plastics One, Roanoke, VA, USA) was unilaterally inserted into the left lateral ventricle in 9-week-old mice. The final coordinates of the cannula tips were as follows: 0.2 mm caudal to bregma, 1.0 mm lateral to the midline, and 2.25 mm ventral to the skull surface. RFRP-3 (6 nmol/day, *n* = 8) was dissolved in saline. For the control animals (*n* = 7), a vehicle solution was employed. The dose of RFRP-3 was determined based on a previous study [58].

The solutions were loaded into an ALZET mini-osmotic pump (delivery rate: 0.25 µL/h, model 1002; DURECT Co., Cupertino, CA, USA) connected to the infusion cannula using polyethylene tubing a day before surgery and were kept overnight at 37 °C. On the day of the cannula insertion, the osmotic pump was implanted subcutaneously into the back. We confirmed that the infusion was correct by examining the solution remaining in the pump at the endpoint.

Food intake and body mass were measured every day. The masses of inguinal, epididymal, retroperitoneal, and perirenal WAT were measured at the endpoint in all of the experiments. The masses of the interscapular BAT, liver, heart, kidney, testis, and gastrocnemius muscle tissues were also measured at the endpoint.

### 4.5. Indirect Calorimetry and Locomotor Activity

Seven to eight days after the infusion of RFRP-3, indirect calorimetry was performed using an O_2_/CO_2_ metabolism-measuring system for small animals (MK-5000RQ; Muromachi Kikai, Tokyo, Japan). The system monitored VO_2_ (mL/min) and VCO_2_ (mL/min) at 3-min intervals and calculated the RQ ratio (VCO_2_/VO_2_). The locomotor activity was simultaneously measured using the SUPERMEX infrared ray passive sensor system (Muromachi Kikai). The measurements were collected hourly or every 15 min over a 24 h period (light period: 09:00 a.m.–21:00 p.m., dark period: 21:00 p.m.–09:00 a.m.) after 24 h of habituation for 7 d after the infusion of RFRP-3. Energy expenditure was calculated using Equation (1) [59]:

(1) energy expenditure (calkgh)=VO2 (mLkgh) × {3.815+(1.232 ×RQ)}. 

### 4.6. qRT-PCR

The hypothalamus, pituitary, iWAT, BAT, and liver were dissected from the mice, snap-frozen in liquid nitrogen, and stored at −80°C for RNA processing at the endpoint of the infusion of RFRP-3. The total RNA was extracted using TRIzol reagent (Life Technologies, Carlsbad, CA, USA) for brain and liver tissues or QIAzol lysis reagent for iWAT and BAT (QIAGEN, Venlo, Netherlands) following the manufacturer’s instructions. The RNA concentration was measured by nanodrop spectroscopy (Thermo Fisher Scientific, Waltham, MA, USA), and the first-strand cDNA was synthesized from the total RNA using a ReverTra Ace kit (TOYOBO, Osaka, Japan). The primer sequences used in this study are listed in Table 2. The PCR amplifications were performed with THUNDERBIRD SYBR quantitative PCR mix (TOYOBO) using the following conditions: 95 °C for 20 s, followed by 40 cycles of 95 °C for 3 s, and 60 °C for 30 s. The PCR products in each cycle were monitored using a Bio-Rad CFX Connect (Bio-Rad Laboratories, Hercules, CA, USA). The relative quantification of each gene was determined by the 2^–∆∆Ct^ method using β-actin (*Actb*) for brain and liver tissues or ribosomal protein S18 (*Rps18*) for iWAT and BAT as internal controls [60].

### 4.7. Blood Chemistry

The serum levels of the glucose, lipids, and hormones were measured using appropriate equipment, reagents, and kits. The GLUCOCARD G+ meter was used to measure the glucose content (Arkray, Kyoto, Japan). The NEFA C-Test (Wako Pure Chemical Industries, Osaka, Japan), the Triglyceride E-Test (Wako Pure Chemical Industries), and the Cholesterol E-Test (Wako Pure Chemical Industries) were used for the free fatty acid levels, triglyceride levels, and cholesterol content, respectively. The LBIS Mouse Insulin ELISA kit U-type (Shibayagi, Gunma, Japan) and the Total Thyroxine ELISA kit (ALPCO, Salem, NH, USA) were used to measure the insulin and T4 levels, respectively.

### 4.8. Statistical Analysis

Group differences between the RFRP-3- and vehicle-treated animals were assessed using Student’s *t*-test. *p* values < 0.05 were considered statistically significant.

## Figures and Tables

**Table 1:** Blood chemistry during chronic intracerebroventricular (i.c.v.) infusion of RFamide-related peptide-3 (RFRP-3).

Serum Components	CTL	RFRP-3
Glucose (mg/dL)	126 ± 7.0	146 ± 6.3*
Free Fatty Acids (mEq/L)	2.25 ± 0.19	1.82 ± 0.26
Triglyceride (mg/dL)	113 ± 10.4	123 ± 13.8
Cholesterol (mg/mL)	99 ± 5.0	104 ± 4.4
Thyroxine (T4) (µg/dL)	0.43 ± 0.18	2.03 ± 0.84
Insulin (ng/mL)	0.77 ± 0.19	0.79 ± 0.10

Each value represents the mean ± standard error of the mean. *n* = 7–8; * *p* < 0.05.

**Table 2:** Sequences of oligonucleotide primers for qRT-PCR.

Gene	Sense Primer (5′ to 3′)	Antisense Primer (5′ to 3′)
*Npy*	TATCTCTGCTCGTGTGTTTG	GATTGATGTAGTGTCGCAGA
*Agrp*	TGTTCCCAGAGTTCCCAGGTC	GCATTGAAGAAGCGGCAGTAGCAC
*Pomc*	AGCTGCCTTTCCGCGACA	ATCTATGGAGGTCTGAAGCA
*Trh*	TCGTGCTAACTGGTATCCCC	CCCAAATCTCCCCTCTCTTC
*Gnrh*	AGCACTGGTCCTATGGGTTG	CCTGGCTTCCTCTTCAATCA
*Rfrp*	TGGAAGGACCATAGATGAGAAA	GCTGTTGTTCTCCCAAACCT
*Gpr147*	CTTTCCGTGAGAAGCTGACC	GAGCATCCAGCATGAAGTGA
*Prl*	GGCTACACCTGAAGACAAGGAACAA	TGTTCCTCAATCTCTTTGGCTCTTG
*Gh*	GGAGGCTAGTGCTTTTCCCG	AGGCACGCTCGAACTCTTTG
*Lhβ*	TGGCCGCAGAGAATGAGTTC	ACTCGGACCATGCTAGGACA
*Fshβ*	GGAGAGCAATCTGCTGCCAT	GCCGAGCTGGGTCCTTATAC
*Tshβ*	CACCATCTGTGCTGGGTATTG	CATCCTGGTATTTCCACCGTTC
*Acc*	TCCGCACTGACTGTAACCACAT	TGCTCCGCACAGATTCTTCA
*Fas*	AGGGGTCGACCTGGTCCTCA	GCCATGCCCAGAGGGTGGTT
*Scd1*	CTGTACGGGATCATACTGGTTC	GCCGTGCCTTGTAAGTTCTG
*Atgl*	AACACCAGCATCCAGTTCAA	GGTTCAGTAGGCCATTCCTC
*Hsl*	GCTGGGCTGTCAAGCACTGT	GTAACTGGGTAGGCTGCCAT
*Pparγ*	GCCCTTTGGTGACTTTATGGA	GCAGCAGGTTGTCTTGGATG
*Pgc1α*	GCAACATGCTCAAGCCAAAC	TGCAGTTCCAGAGAGTTCCA
*Ucp1*	CAAAAACAGAAGGATTGCCGAAA	TCTTGGACTGAGTCGTAGAGG
*Dio2*	CCACCTTCTTGACTTTGCCA	GGTGAGCCTCATCAATGTATAC
*Cidea*	CTTATCAGCAAGACTCTGGATG	GAAGGTGACTCTGGCTATTC
*Cox4*	TGAGCCTGATTGGCAAGAGA	CGAAGCTCTCGTTAAACTGG
*Gpat1*	TCATCCAGTATGGCATTCTCACA	GCAAGGCCAGGACTGACATC
*Chrebpα*	CGACACTCACCCACCTCTTC	TTGTTCAGCCGGATCTTGTC
*Chrebpβ*	TCTGCAGATCGCGTGGAG	CTTGTCCCGGCATAGCAAC
*Cpt1a*	CCTGGGCATGATTGCAAAG	GGACGCCACTCACGATGTT
*Gapdh*	AAGGTCATCCCAGAGCTGAA	CTGCTTCACCACCTTCTTGA
*Cd36*	TCCTCTGACATTTGCAGGTCTATC	AAAGGCATTGGCTGGAAGAA
*Pepck*	GTGCTGGAGTGGATGTTCGG	CTGGCTGATTCTCTGTTTCAGG
*G6pase*	ACTGTGGGCATCAATCTCCTC	CGGGACAGACAGACGTTCAGC
*Slc2a2*	GGCTAATTTCAGGACTGGTT	TTTCTTTGCCCTGACTTCCT
*Pparα*	TCGAATATGTGGGGACAAGG	GACAGGCACTTGTGAAAACG
*Tnfα*	GCCTCTTCTCATTCCTGCTTG	CTGATGAGAGGGAGGCCATT
*Slc2a4*	GTAACTTCATTGTCGGCATGG	AGCTGAGATCTGGTCAAACG
*Actb*	GGCACCACACCTTCTACAAT	AGGTCTCAAACATGATCTGG
*Rps18*	CCTGAGAAGTTCCAGCACAT	TTCTCCAGCCCTCTTGGTG

## Supplementary Materials

The following are available online at https://www.mdpi.com/1422-0067/21/22/8606/s1, Figure S1: The effects of chronic i.c.v. infusion of RFRP-3 on the mRNA expression of feeding and energy metabolism-related genes in the hypothalamus and pituitary; Figure S2: The effects of chronic i.c.v. infusion of RFRP-3 on the mRNA expression of lipid metabolism-related genes in the adipose tissues (BAT and iWAT) and liver, and thermogenesis-related genes in the BAT.

## Abbreviations

ACC	Acetyl-CoA carboxylase
ACTB	β-actin
AgRP	Agouti-related peptide
Arc	Arcuate nucleus
ATGL	Adipose triglyceride lipase
BAT	Brown adipose tissue
CD36	Cluster of differentiation 36
ChREBPα, β	Carbohydrate-responsive element-binding protein α, β
CIDEA	Cell death-inducing DNA fragmentation factor-like effector A
COX4	Cytochrome c oxidase subunit 4
CPT1a	Carnitine palmitoyltransferase 1a
DIO2	Type II iodothyronine deiodinase
DMH	Dorsomedial hypothalamus
FAS	Fatty acid synthase
FSH	Follicle-stimulating hormone
G6Pase	Glucose-6-phosphatase
GAPDH	Glyceraldehyde-3-phosphate dehydrogenase
GH	Growth hormone
GnIH	Gonadotropin-inhibitory hormone
GnRH	Gonadotropin-releasing hormone
GPAT1	Glycerol-3-phosphate acyltransferase 1
GPR147	G protein-coupled receptor 147
HPG	Hypothalamic–pituitary–gonadal
HSL	Hormone-sensitive lipase
i.c.v.	Intracerebroventricular
iWAT	Inguinal white adipose tissue
LH	Luteinizing hormone
NPY	Neuropeptide Y
PEPCK	Phosphoenolpyruvate carboxykinase
PGC1α	Peroxisome proliferator-activated receptor γ coactivator 1α
POMC	Proopiomelanocortin
PPARα, γ	Peroxisome proliferator-activated receptor α, γ
PRL	Prolactin
PVN	Paraventricular nucleus
RFRP	RFamide-related peptide
RPS18	Ribosomal protein S18
RQ	Respiratory quotient
SCD1	Stearoyl-CoA desaturase 1
SLC2A	Solute carrier family 2 member
T4	Thyroxine
TNFα	Tumor necrosis factor α
TRH	Thyrotropin-releasing hormone
TSH	Thyroid-stimulating hormone
UCP1	Uncoupling protein 1
VCO_2_	CO_2_ production
VO_2_	O_2_ consumption
WAT	White adipose tissue
