# Supplementary material for: Effects of Chronic Intracerebroventricular Infusion of RFamide-Related Peptide-3 on Energy Metabolism in Male Mice
Source: Int J Mol Sci. 2020 Nov 15;21(22):8606. doi: 10.3390/ijms21228606 (PMC7698077; doi:10.3390/ijms21228606)
Supplement: Supplementary file 1 [file ijms-21-08606-s001.pdf]

International Journal of
Molecular Sciences

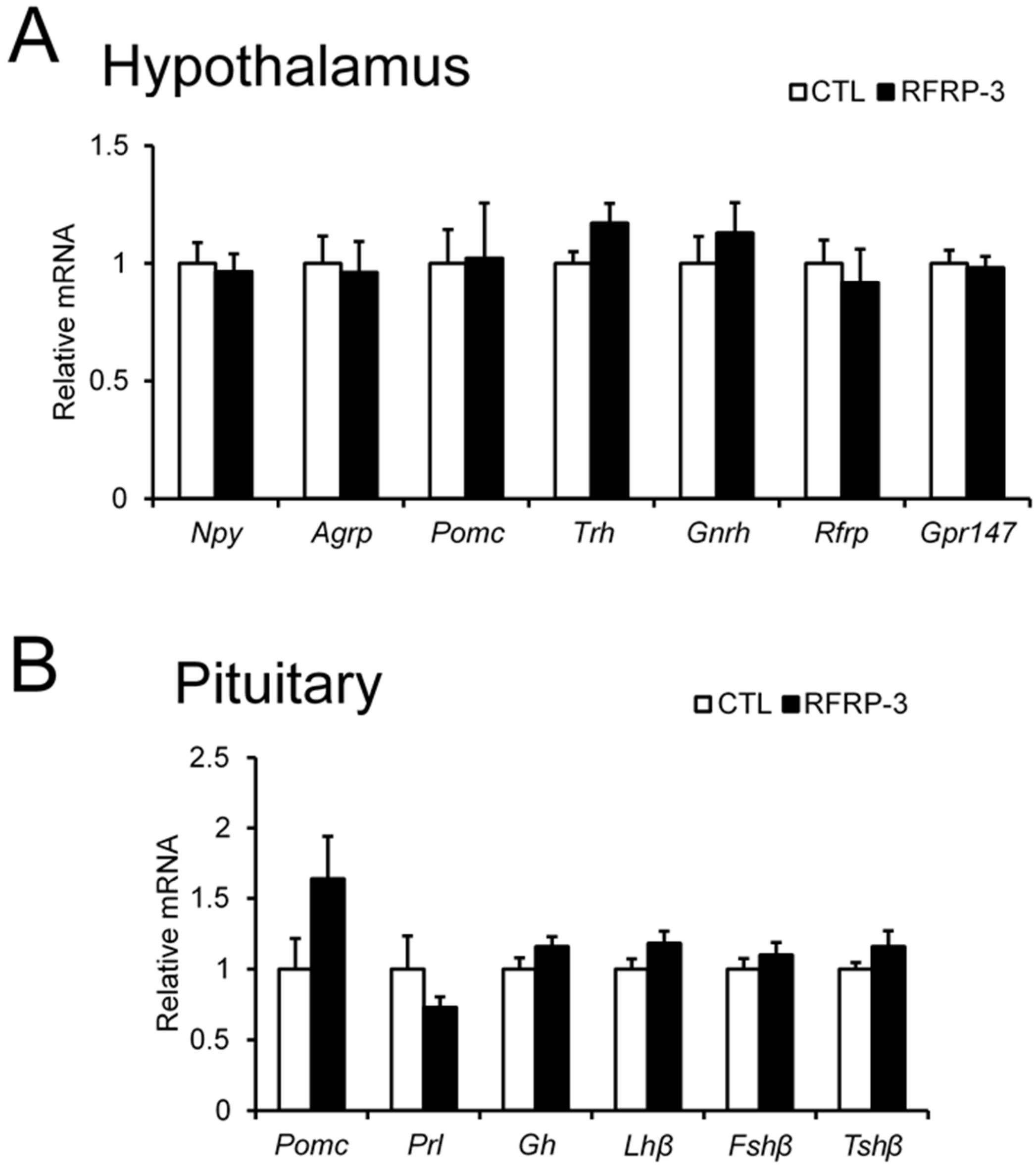

Figure S1. The effects of chronic i.c.v. infusion of RFRP-3 on the mRNA expression of feeding and energy metabolism-related genes. mRNA expression levels for (**A**) hypothalamus and (**B**) pituitary. We chose neuropeptide Y (NPY), agouti-related peptide (AgRP), and proopiomelanocortin (POMC) as neuropeptides related to feeding; thyrotropin-releasing hormone (TRH), growth hormone (GH), and thyroid-stimulating hormone β (TSHβ) as neuropeptides related to energy metabolism; gonadotropin-releasing hormone (GnRH), prolactin (PRL), luteinizing hormone β (LHβ), and follicle-stimulating hormone β (FSHβ) as neuropeptides related to reproduction; RFRP and G protein-coupled receptor 147 (GPR147), the receptor for RFRP-3. Each value represents the mean ± standard error of the mean. $n = 7–8$.

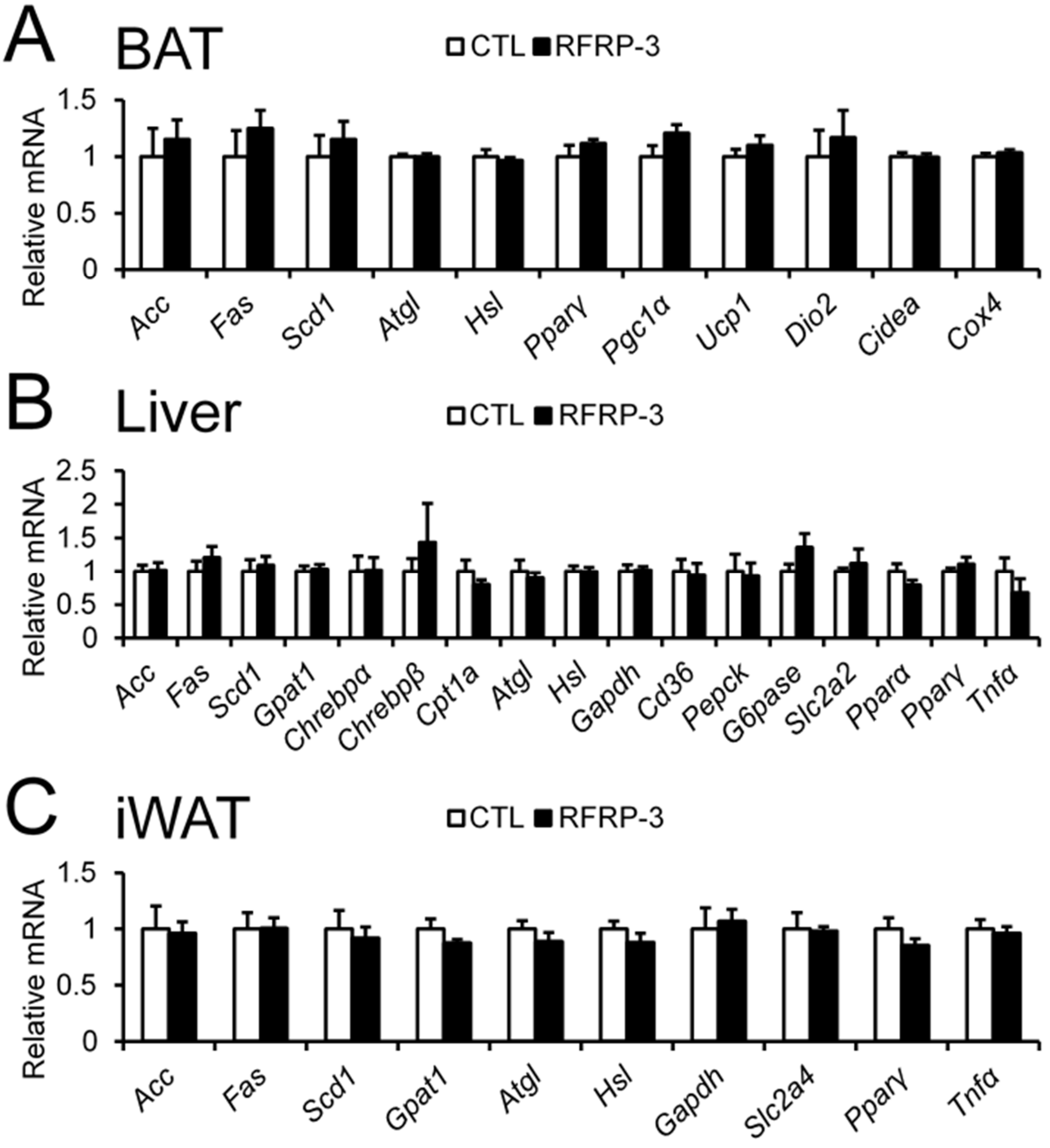

Figure S2. The effects of chronic i.c.v. infusion of RFRP-3 on the mRNA expression of lipid metabolism-related genes in the adipose tissues and liver, and thermogenesis-related genes in the BAT. mRNA expression levels for (**A**) BAT, (**B**) liver, and (**C**) inguinal WAT (iWAT). We chose acetyl-CoA carboxylase (ACC), fatty acid synthase (FAS), stearoyl-CoA desaturase 1 (SCD1), and glycerol-3-phosphate acyltransferase 1 (GPAT1) as lipogenic enzymes; carbohydrate-responsive element-binding protein α, β (ChREBPα, β) as lipogenic transcription factors; carnitine palmitoyltransferase 1a (CPT1a), adipose triglyceride lipase (ATGL), and hormone-sensitive lipase (HSL) as lipolytic enzymes; peroxisome proliferator-activated receptor α, γ (PPARα, γ) as lipid-activated transcription factors; glyceraldehyde-3-phosphate dehydrogenase (GAPDH) as a carbohydrate metabolism gene; cluster of differentiation 36 (CD36) as a fatty acid transporter; phosphoenolpyruvate carboxykinase (PEPCK) and glucose-6-phosphatase (G6Pase) as gluconeogenesis enzymes; solute carrier family 2 member 2 (SLC2A2) and solute carrier family 2 member 4 (SLC2A4) as glucose transporters; tumor necrosis factor α (TNFα) as an inflammatory cytokine; peroxisome proliferator-activated receptor γ coactivator 1α (PGC1α), uncoupling protein 1 (UCP1), type II iodothyronine deiodinase (DIO2), cell death-inducing DNA fragmentation factor-like effector A (CIDEA), and cytochrome c oxidase subunit 4 (COX4) as genes related to thermogenic function. Each value represents the mean ± standard error of the mean. n = 7–8.
